# Noise Parameters of Headsets Designed for Communication Platforms

**DOI:** 10.3390/ijerph19063369

**Published:** 2022-03-12

**Authors:** Emil Kozlowski

**Affiliations:** Department of Vibroacoustic Hazards, Central Institute for Labour Protection—National Research Institute, Czerniakowska 16, 00-701 Warsaw, Poland; emkoz@ciop.pl; Tel.: +48-22-623-3294

**Keywords:** occupational exposure, sound, noise, headsets, sound pressure level

## Abstract

Headsets are increasingly used in the working environment. In addition to being frequently used by call-centre staff, they are also becoming more popular with remote workers and teleconference participants. The aim of this work was to describe and evaluate the acoustic signal parameters reproduced by headsets and examine the factors affecting the values of these parameters. The tests were carried out in laboratory conditions using a manikin (head and torso simulator) designed for acoustic research. A total of 12 headset models were tested during the research. The results show that the A-weighted sound pressure level of the test signal reproduced by four (100% gain) and two (75% gain) headsets exceeded 85 dB. The highest equivalent A-weighted sound pressure level was 92.5 dB, which means that the headset should not be used for more than approx. 1 h and 25 min; otherwise, the criterion value will be exceeded. The analysis of the acoustic signal reproduced by the headsets confirmed that the A-weighted sound pressure level affected the gain level in the test signal reproduction path. This value also depended on the type of connector used, the computer from which the test signal was reproduced and the type of sound card used.

## 1. Introduction

In recent years, and especially over the last two years, headsets have been used more frequently in verbal communication due to the shift towards remote working caused by (among other factors) the COVID-19 pandemic. For instance, headsets are widely used to communicate during teleconferences through internet platforms. Headsets are also used by call-centre, airport-ground-handling and air-traffic-control staff.

Noise measurements are typically carried out according to ISO 9612:2009 [1] using sound level meters or noise dosimeters. However, this standard does not cover situations where the noise source is placed close to the ear of the worker. In this case, noise parameters must be measured using a miniature microphone, which is inserted in the human ear (MIRE technique), in accordance with ISO 11904-1:2002 [2], or a manikin designed for acoustic research, as set out in ISO 11904-2:2021 [3]. The standards ISO 11904-1:2002 [2] and ISO 11904-2:2021 [3] define a method for determining the free-field or diffuse-field related sound pressure level in cases where the source of noise is placed close to the ear using free-field or diffuse-field frequency responses defined in 1/3-octave bands. With this method, some assessment criteria for noise exposure as specified in relevant documents can be used for workplaces where the noise source is placed close to the ear. In Poland, the following three parameters are taken into account in the evaluation of exposure to occupational noise: the daily noise exposure level (L_EX,8h_), the A-weighted maximum sound pressure level (L_Amax_), and the C-weighted peak sound pressure level (L_Cpeak_). The daily noise exposure level, L_EX,8h_, is calculated from the A-weighted equivalent sound pressure level (L_Aeq_) and the exposure duration. The L_EX,8h_ should not exceed 85 dB; the L_Amax_, 115 dB; and the L_Cpeak_, 135 dB [4]. The above assessment system differs from that specified in Directive 2003/10/EC [5], which defines the upper exposure action values as 85 dB for L_EX,8h_ and 137 dB for L_Cpeak_, and the exposure limit values are L_EX, 8h_ = 87 dB and L_Cpeak_ = 140 dB. Furthermore, the above directive, unlike the Polish laws, does not provide an assessment for L_Amax_.

In addition to the aforementioned methods for determining the sound pressure level in cases where the noise source is placed close to the ear using a miniature microphone [2] and a manikin [3], researchers from New Zealand, Australia and Canada have developed a test method that replaces the manikin and the miniature microphone with an artificial ear. This method is described in AS/NZS 1269.1:2005 [6], CSA Z107.56-13 [7] and CSA Z107.56-18 [8]. AS/NZS 1269.1:2005 [6] and CSA Z107.56-13 [7] differ from ISO 11904-1:2002 [2] and ISO 11904-2:2021 [3] in that they define single-number corrections that are used to convert measurement results into free-field or diffuse-field levels.

A number of studies on the acoustic signal parameters reproduced by headsets have been carried out. The available literature indicates that there is a significant disparity between the results of the noise parameter tests under headsets. When headsets are used, the A-weighted equivalent sound pressure level ranges from approx. 50 to over 100 dB [9,10,11,12,13,14,15,16,17,18,19,20]. Such a disparity between test results is due to the different measurement methods, headsets, measurement conditions (background noise making it necessary to increase the gain of an acoustic signal) or workplaces used in the testing process. Another reason for such disparity is the wide sound level variability within the same study, which is due to the specific type of the acoustic signal generated by headsets. This means that quieter moments (no acoustic signal being reproduced by the headsets) and louder moments (resulting from the reproduction of a speech signal and short-term signals, such as telephone disruptions) may occur interchangeably during the measurements. Studies published in the scientific literature show that noise exposure from headset use may be associated with some risk of hearing damage [15,21,22,23,24]. There are also studies that demonstrate that noise exposure from using headsets presents little or no risk of hearing loss [19,25,26,27].

The aim of this work was to describe and evaluate the acoustic signal parameters reproduced by headsets and examine the factors affecting the values of these parameters.

In this study, measurements were taken in laboratory conditions, so that measurements could be made for a large number of headsets and so that the effects of various factors—such as the model of computer, the use of an external sound card, the volume control level of a signal and the type of connector—on the sound pressure level that reaches the person using the headset could be analysed. In addition, the latest available manikin was used. This manikin allows measurements within the full range of human hearing.

## 2. Materials and Methods

### 2.1. Headsets

Twelve models of headsets from four different manufacturers were included in the study. The selected headsets are commonly used in call centres and by employees participating in teleconferences. These included 5 headsets with only a USB connector (designated A, B, C, K and L for the purposes of this study), one headset with only a 3.5 mm-jack connector (designated D), 5 universal headsets with both connectors (designated E, F, G, H and I) and one headset that used Bluetooth technology for signal transmission (designated L). The mentioned designations are used later in this article in the section in which tables and graphs with the test results are presented.

### 2.2. Measuring Equipment

The measurements were carried out using a manikin designed for acoustic research, the Brüel&Kjær High-frequency Head and Torso Simulator Type 5128 (Hottinger Brüel&Kjær A/S, Virum, Denmark). This manikin is designed to allow measurements within the full range of human hearing (20–20,000 Hz). The manikin consists of a head with an ear simulator and torso. These parts represent the average dimensions of an adult human. The ear simulator consists of a removable silicon-rubber pinna connected to a human-like ear canal. A measuring microphone and a microphone preamplifier are placed at the end of the ear canal. The pinna of the manikin meets the requirements of ITU-T Rec. P.57 [28] and is designed to have a hardness of 35 on the Shore-00 scale. The shape and stiffness of the pinna allow for realistic deformations in the headphone elements used for the measurements.

**Figure 1:**
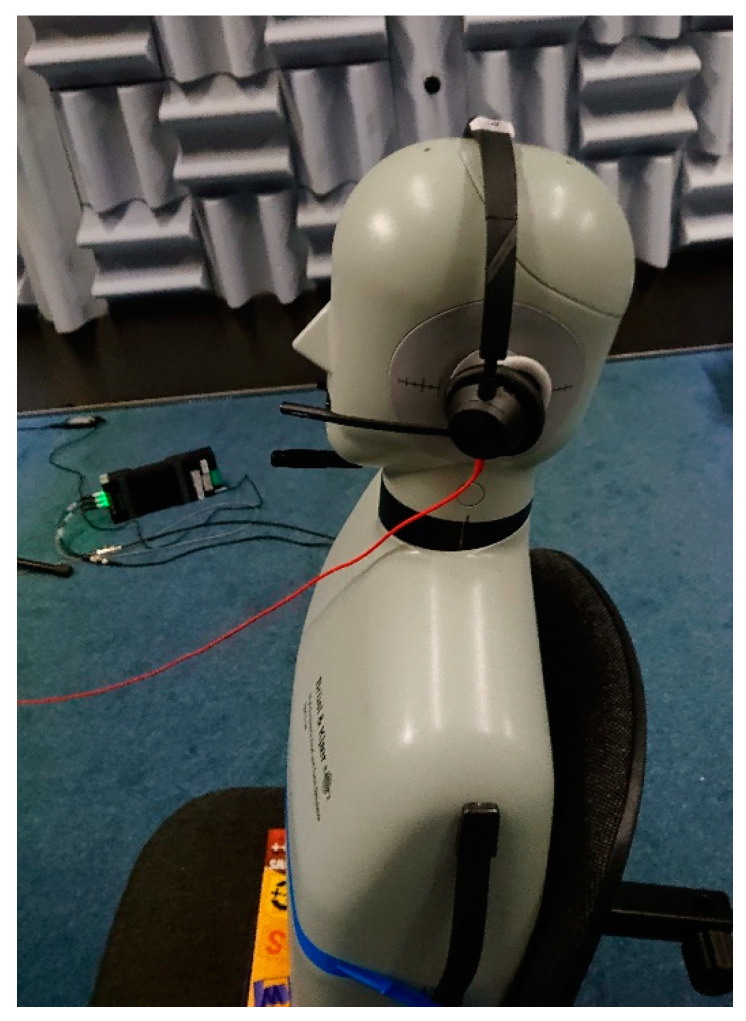
Measurement system for the noise parameters generated by headsets. On the left, the Brüel&Kjær PULSE 3052-A-030 030 (Hottinger Brüel&Kjær A/S, Virum, Denmark) measurement module is shown in the background; the acoustic manikin Brüel&Kjær High-frequency Head and Torso Simulator Type 5128 (Hottinger Brüel&Kjær A/S, Virum, Denmark) with attached headset is shown in the foreground.

The measuring module Brüel&Kjær PULSE 3052-A-030 (Hottinger Brüel&Kjær A/S, Virum, Denmark), connected to the manikin, along with the Brüel&Kjær PULSE Time Data Recorder software (Hottinger Brüel&Kjær A/S, Virum, Denmark), was used to record the acoustic signal reproduced by the headset. The measurements were carried out under free-field conditions. A photo of the measuring system is shown in Figure 1.

### 2.3. Test Signal

The test signal was a speech sound uttered by a man. It was a fragment of a 1 min staged conversation made via communication platform installed on two computers. Such a simulated situation may apply both to the situation of a consultant’s conversation with a customer and to a conversation during a teleconference.

One computer was connected to a headset with a microphone that picked up the speech signal. This signal reached the second computer via the communication platform. The speech signal was recorded as a *.wav file using the Brüel&Kjær PULSE 3052-A-030 (Hottinger Brüel&Kjær A/S, Virum, Denmark) measurement module, controlled by the Brüel&Kjær PULSE Time Data Recorder (Hottinger Brüel&Kjær A/S, Virum, Denmark) software. The measurement module was directly connected to the sound card output of the second computer used for the conversation. In addition to words, the recorded conversation contained the artefacts likely to occur in real-life talk, i.e., the crackles resulting from the headset being adjusted to better fit the head and accidental contact between the hand and the microphone.

The test signal recorded as described above was played using a sound player built into the operating system, which was installed on the computer to which the headsets placed on the manikin were connected.

Two different computers were used in the tests: a Lenovo ThinkPad T440p (Lenovo Group Ltd., Beijing, China), designated Laptop 1 for the purposes of this study, and a Dell Latitude 5580 (Dell, Round Rock, TX, USA), designated Laptop 2, for the purposes of this study. In addition, for Laptop 1, a mobile audio interface, ESI MAYA 44 USB (ESI Audiotechnik GmbH, Leonberg, Germany), was used instead of the sound card integrated into the computer’s motherboard.

### 2.4. Test Method

Each of the 12 headsets included in the study was placed on the head of the manikin, one after another. Then, a test signal was generated from Laptop 1. The signal was generated three times—for volume control levels of 75%, 90% and 100%. The level of 75% represents the subjective perception of that at which good audibility of the test signal is achieved for all the measurement situations. The level of 100% represents the situation of the greatest possible exposure to sound. The level of 90% is an intermediate setting between the two previous situations. The headset was connected to the computer via a USB port or a 3.5 mm jack. For headsets equipped with both types of connectors, the measurement was carried out (in both cases) without removing the headset from the manikin’s head. A headset connected to the computer via wireless Bluetooth was also used. The recording of the acoustic signal reproduced by the headset started after the test signal was triggered. This recording was performed using the microphones of the manikin connected to the measurement module. Then, the BK Connect 2019 (Hottinger Brüel&Kjær A/S, Virum, Denmark) software was used to establish the sound pressure level (SPL) in 1/3-octave bands, the L_Aeq_, the L_Amax_ and the L_Cpeak_. The L_Aeq_ was converted in accordance with ISO 11904-2:2021 [3]. The above standard sets out a method for determining the A-weighted equivalent sound pressure level from sources placed close to the ear through the use of a manikin, under which the measured values are converted into corresponding free-field or diffuse-field levels. This method makes use of experimentally determined free-field or diffuse field frequency responses for use with manikins provided in the mentioned standard ISO 11904-2:2021 [3]. This response is provided in the 1/3-octave band centre frequency. The values of the SPL measured with the use of the manikin are corrected according to the mentioned response. Then, the L_Aeq_ is calculated on the basis of the corrected values of the SPL.

The standard ISO 11904-2:2021 [3] does not specify how to convert the L_Amax_ and L_Cpeak_ values measured using a manikin so that they reflect the free-field or diffuse-field related levels. It can be suspected that this conversion for L_Amax_, may take the same values as for L_Aeq_. It may be different for L_Cpeak_, due to the use of different weighting characteristics in this parameter. This paper is part of a larger ongoing study to develop a method for determining L_Amax_ and L_Cpeak_ from sources placed close to the ear so that measured values can be related to the conditions of free-field or diffuse-field levels. At this stage, the study was carried out in free-field conditions, with the use of free-field frequency response therefore this response was also used in this paper.

The same procedure was followed for measurements and calculations that involved a test signal reproduced using Laptop 2 and when a mobile audio interface was connected to Laptop 1. Due to the type of outputs used in this interface, i.e., RCA outputs, the measurements were carried out using six headsets equipped with a 3.5 mm jack.

### 2.5. Statistical Analysis

In order to determine whether the use of different models of computers to which the headsets were connected and the type of connector used affected the L_Aeq_ reproduced by these headsets, statistical analyses involving a parametric *t*-test and a non-parametric Wilcoxon test were carried out. The calculations were performed using MATLAB R2010b version 7.11.0.584 (MathWorks Inc., Natick, MA, USA).

## 3. Results

### 3.1. Evaluation of Noise Exposure

**Figure 2:**
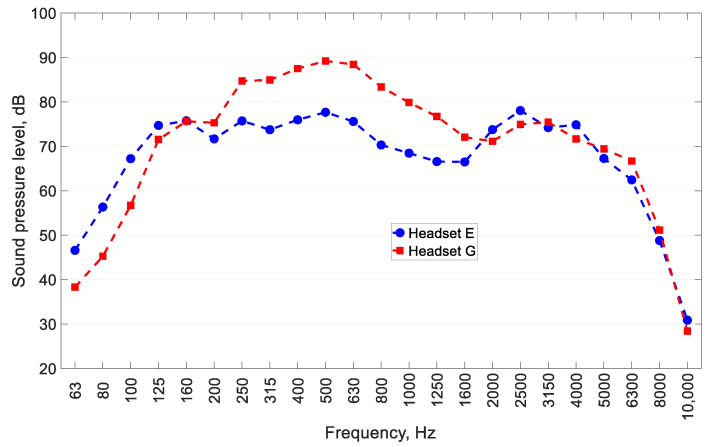
Sound pressure level in 1/3-octave bands of the test signal reproduced by Headsets E and G connected to Laptop 2.

Table 1, Table 2 and Table 3 show the properly measured values for the A-weighted equivalent sound pressure level, the A-weighted maximum sound pressure level and the C-weighted peak sound pressure level for the test signals reproduced by different types of headsets. Table 1, Table 2 and Table 3 show the higher values of the parameters measured in the left and right ears of the manikin. The values for L_Aeq_ presented in Table 1 were converted as set out in ISO 11904-2:2021 [3] to correspond to the SPL in free-field conditions. As a result of this conversion, the L_Aeq_ was reduced by 2.4 to 10.8 dB, depending on the headset used. For these extreme values, the headsets affected by the conversion were those designated as G (USB connector) and E (3.5 mm-jack connector), respectively. The change in L_Aeq_ resulting from this type of conversion is due to the values of the SPL in the 1/3-octave bands of the test signal. In the case of Headset G, it can be observed that the SPL in the 1/3-octave bands of the test signal (Figure 2) reached its highest values at low frequencies (250–630 Hz). Therefore, the conversion did not significantly affect the L_Aeq_ (2.4 dB). However, the analysis of the SPL in the 1/3-octave bands of the test signal for Headset E, as shown in Figure 2, shows that the spectrum was flat. This means that the L_Aeq_ was, to a greater extent, affected by the conversion (10.8 dB).

The use of the free-field frequency response allows for the limit values in the working environment to be addressed more directly. As mentioned earlier, the limit value of L_EX, 8h_ is 85 dB in Poland [4]. The results of the L_Aeq_ measurements can be used to calculate the duration of exposure to the acoustic signal so that the aforementioned criterion value is not exceeded.

Table 1 shows the L_Aeq_ of the test signal reproduced by all the headsets at a volume control level of 100%. Table 1 also lists the L_Aeq_ values for the test signal at a volume control level of 90% but only if L_Aeq_ exceeds 85 dB. The same procedure was followed for the test signal at a volume control level of 75%.

The obtained L_Aeq_ values of the test signal at a volume control level of 100% ranged from 64.2 to 92.5 dB. For 4 out of the 12 headsets being tested, L_Aeq_ values above 85 dB were obtained. Under these conditions, given that the exposure duration is sufficiently long, the limit value of L_EX,8h_ is likely to be exceeded. The highest L_Aeq_ of the test signal was identified for Headset G. When this headset was connected to the sound cards integrated with Laptop 1 or Laptop 2, the L_Aeq_ of the test signal would be above 85 dB, regardless of the connector used (USB, 3.5 mm jack or Bluetooth). The highest value of 92.5 dB obtained for this headset means that the exposure duration cannot be longer than 1 h and 25 min.

When the signal volume control was reduced to 90%, it was also observed that the L_Aeq_ value of 85 dB was exceeded for four headsets. Unlike when the volume control level was set to 100%, this only applied to headsets with a 3.5 mm-jack connector and Bluetooth interface. When the signal volume control was set to 75%, the L_Aeq_ value 85 dB was exceeded for two headsets (Laptop 2; 3.5 mm-jack connector).

Additionally, it should be noted that the same headsets, at a given volume control level, reproduce different SPLs, depending on whether they are connected via USB or 3.5 mm-jack connectors. This is naturally related to the fact that the use of different connectors means the use of different digital-to-analogue converters, i.e., embedded in the laptop (in case of the 3.5 mm-jack connector) or integrated with the headsets (in case of the USB connector).

When the headsets were connected to Laptop 1 via a mobile audio interface, no L_Aeq_ above 85 dB was identified for any of the headsets used. This means that, with the sound reproduction system and test signal described above, these headsets can be used for the entire eight-hour shift without the risk of damage to hearing.

Table 2 and Table 3 show, respectively, the L_Amax_ and L_Cpeak_ of the test signal reproduced by various headsets that were directly connected to Laptop 1 and Laptop 2, and to Laptop 1 through a mobile audio interface. The results shown in Table 2 and Table 3 correspond to a volume control level of 100% for the test signal. The standard ISO 11904-2:2021 [3] does not specify how to convert the L_Amax_ and L_Cpeak_ values measured using a manikin so that they reflect the free-field related levels. Therefore, the values obtained by means of the manikin without any conversion were further examined. For headsets connected to the laptops directly, the L_Amax_ values ranged from 84.8 to 99.8 dB. When a mobile audio interface was used, the observed L_Amax_ values of the reproduced test signal were lower than those with the headsets directly connected to computers, and ranged from 76.2 to 86.3 dB. A comparison of the obtained L_Amax_ values against the limit value applicable in Poland, i.e., 115 dB [4], shows that this value was not exceeded in any of the above scenarios.

The L_Cpeak_ values presented in Table 3 ranged from 106.4 to 116.6 dB when the headsets were connected directly to the laptops. As was the case with the L_Aeq_ and L_Amax_ parameters, the L_Cpeak_ of the test signal when a mobile audio interface was used was lower than that when the headsets were connected directly to computers, and ranged from 96.1 to 105.6 dB. A comparison of the obtained L_Cpeak_ values against the limit value applicable in Poland, i.e., 135 dB [4], shows that, as was the case with L_Amax_, this limit was not exceeded in any of the above scenarios.

### 3.2. Analysis of the Parameters of the Test Signal Reproduced by Headsets in Terms of Volume Control Level, Connector Type and Computer Model

**Figure 3:**
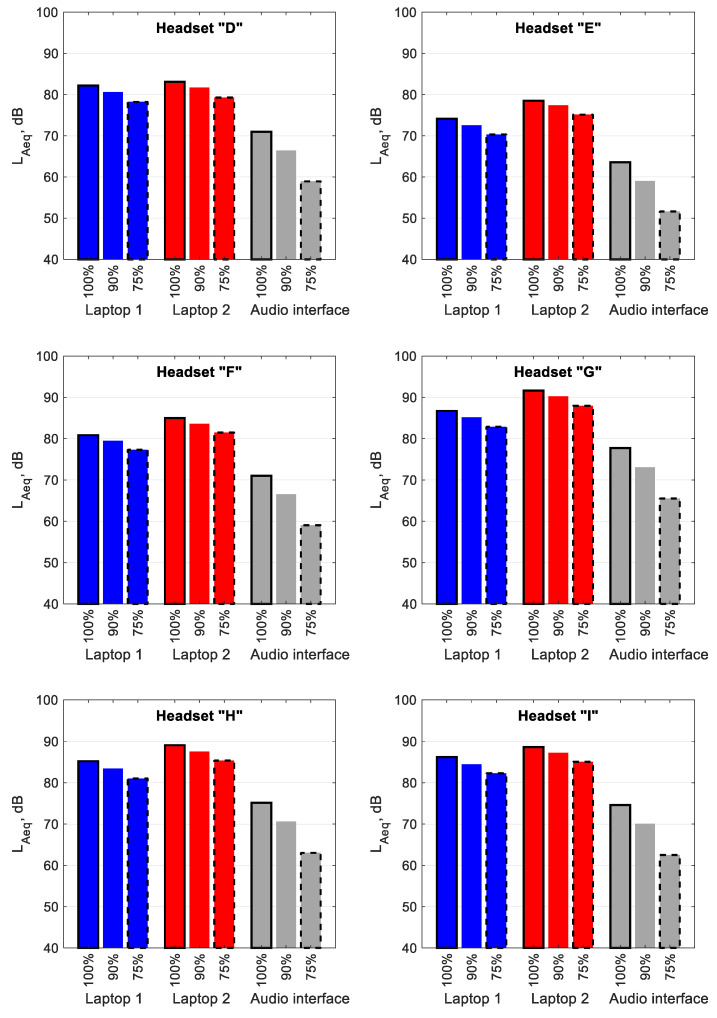
The values of A-weighted equivalent sound pressure level of the test signal reproduced by headsets connected to Laptop 1 and Laptop 2 as well as by headsets connected to Laptop 1 through a mobile audio interface using a 3.5 mm-jack connector. The values given as percentages indicate the control level values of the test signal.

Figure 3 shows the average L_Aeq_ values (the mean values from measurements in the left and right ears of the manikin) of the signal reproduced by the headsets directly connected to Laptop 1 (blue bars) and Laptop 2 (red bars), as well as to Laptop 1 via a mobile audio interface (grey bars) using a 3.5 mm-jack connector. As the volume control level of the test signal decreased, so did the L_Aeq_. For headsets directly connected to computers, these L_Aeq_ changes were not very significant and averaged around 1.5 dB (when the signal volume control level changed from 100 to 90%) and around 2 dB (when the signal volume control level changed from 90 to 75%), regardless of the headset and laptop model. More significant changes related to the signal volume control level in L_Aeq_ were observed when headsets were connected to Laptop 1 through a mobile audio interface. The changes in L_Aeq_ amounted to 4.5 and 7.5 dB, on average, when the signal volume control level was reduced from 100 to 90% and from 90 to 75%, respectively.

Table 4 shows the mentioned changes in the average L_Aeq_ of the test signal and changes in the equivalent electrical signal level at the output of the sound cards due to changes in the volume control level. Measurements upon changes in the volume control level from 100 to 90% and from 90 to 75% show that changes in the acoustic signal correspond to changes in the electrical signal.

Additionally, in the case of the electrical signal, measurements were conducted upon changes in the volume control level in successive ranges of 25%, i.e., from 100 to 75%, from 75 to 50% and from 50 to 25%. The changes in the electric signal in the case of the mobile audio interface were uniform, i.e., the interface worked linearly in the analysed range, whereas in the case of the laptops, the changes were not proportional to the changes in the volume control level. This had a direct impact on the L_Aeq_ at particular volume control level settings.

**Figure 4:**
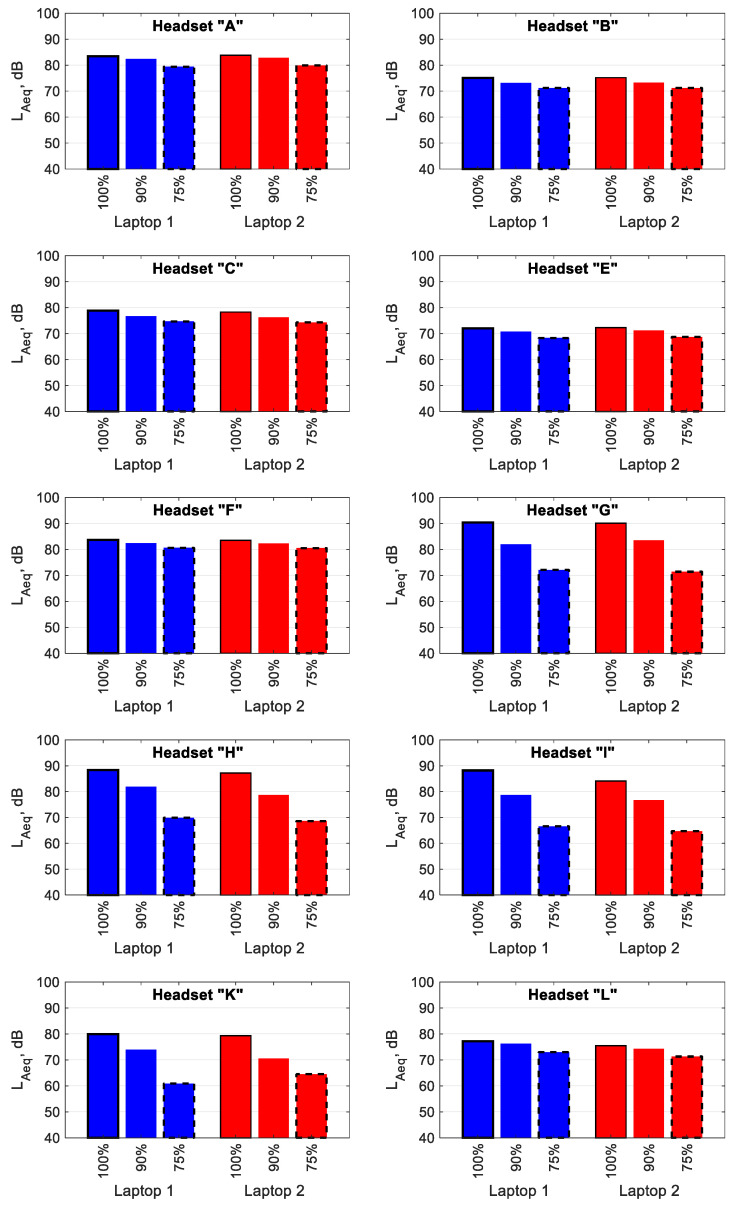
The values of A-weighted equivalent sound pressure level of the test signal reproduced by headsets connected to Laptop 1 and Laptop 2 via USB. The values given as percentages indicate the control level values of the test signal.

Figure 4 shows the mean L_Aeq_ values of the signal reproduced by the headsets connected to Laptop 1 (blue bars) and Laptop 2 (red bars) via USB. On average, a reduction in the L_Aeq_ of approx. 4 dB as a result of the signal volume control level being lowered from 100 to 90% can be observed, and that of approx. 6 dB as a result of the signal volume control level being reduced from 90 to 75% can be observed. However, unlike when a 3.5 mm-jack connector is used, the above changes vary in value depending on the headset model used and range from 1 to 9.5 dB (a reduction from 100 to 90%) and from 1.8 to 13 dB (a reduction from 90 to 75%). The small changes in L_Aeq_ apply to Headsets A, B, C, E, F and L. The large changes in L_Aeq_ apply to Headsets G, H, I and K.

**Figure 5:**
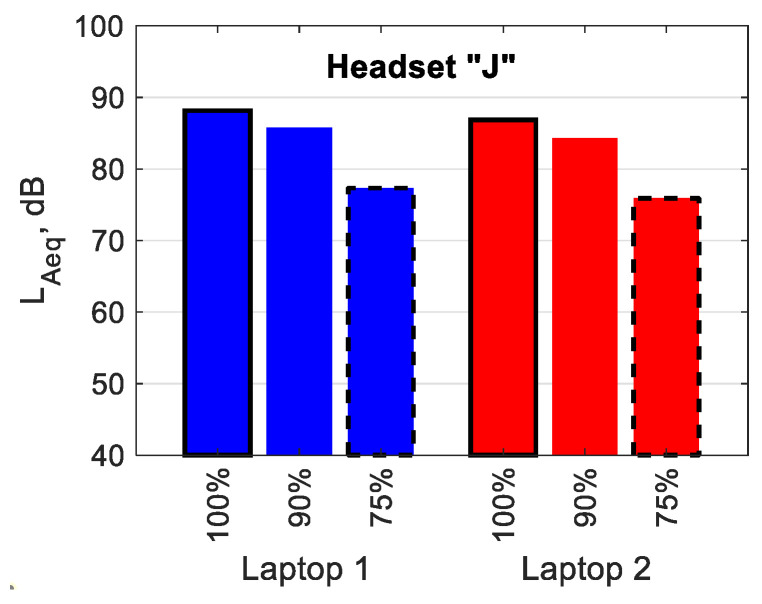
The values of A-weighted equivalent sound pressure level of the test signal reproduced by Headset J connected to Laptop 1 and Laptop 2 via Bluetooth. The values given as percentages indicate the control level values of the test signal.

Figure 5 shows the mean L_Aeq_ values of the signal reproduced by Headset J connected to Laptop 1 (blue bars) and Laptop 2 (red bars) using Bluetooth. A reduction in the L_Aeq_ of approx. 2 dB when the signal volume control level was changed from 100 to 90% can be observed, and that of approx. 8 dB when the signal volume control level was changed from 90 to 75% can be observed, regardless of the computer used.

The reason that low reductions in the L_Aeq_ values for headsets connected using a 3.5 mm-jack were obtained, after changing the test signal volume control level, was the low dynamic range of the sound cards used in the computers. The dynamic range was much higher for the mobile audio interface, which directly contributed to more significant changes in L_Aeq_. For headsets connected to computers via USB or Bluetooth, the dynamic range was both small and large due to the fact that the individual headsets were equipped with different digital-to-analogue converters.

A comparison of the L_Aeq_ values of the test signal reproduced through the headsets connected to two different computers reveals small differences between these values when a 3.5 mm-jack connector was used. The general tendency is that the L_Aeq_ values observed for the headsets connected to Laptop 2, at a signal volume control level of 100%, 90% and 75%, are, on average, 3.4, 3.7 and 3.7 dB higher (respectively) than those obtained for the headsets connected to Laptop 1. A statistical analysis carried out at a confidence level of α = 0.05, involving a parametric *t*-test or a non-parametric Wilcoxon test, revealed that in none of these cases were these changes statistically significant (*p* = 0.23, *p* = 0.2 and *p* = 0.2 for each signal volume control level, respectively).

For headsets connected to Laptop 1 via a mobile audio interface, statistically significant changes in L_Aeq_ could be observed compared to when the headsets were connected directly to the computer. The use of a mobile audio interface resulted in the L_Aeq_ being reduced by 10.4, 13.3 and 18.6 dB, at signal volume control levels of 100%, 90% and 75%, respectively, compared to when Laptop 1 with the sound card integrated into the computer motherboard was used. A statistical analysis revealed a statistically significant difference in the L_Aeq_ values of the test signal, with *p* values of 0.004, 7 × 10^−4^ and 5 × 10^−^5, respectively, for each signal volume control level.

For the test signal reproduced by headsets connected to two different computers, small differences between the L_Aeq_ values were observed when the USB connector was used. A statistical analysis showed that the L_Aeq_ values of the test signal reproduced by different headsets, at volume control levels of 100%, 90% and 75%, did not statistically significantly differ, with *p* values of 0.78, 0.69 and 0.95 for each signal volume control level, respectively. This is because it is not the computers that directly affected the level of the test signal, but the sound cards used in the headsets (analogue-to-digital converters).

## 4. Discussion

This study was conducted to identify the potential sound levels of the acoustic signal reaching the ear of the headset user. It showed that, for certain headset models, there is a risk that, under certain conditions, the sound reaching the headset user may exceed the limit value of 85 dB [4]. This means that there is a risk of hearing damage to the headset user exposed to the sound under test for a long time. Moreover, the study describes the impact of the following factors on the L_Aeq_: the type of connector used, signal volume control level, model of the computer used to reproduce the sound and use of an external sound card. The tests were carried out in laboratory conditions using a manikin designed for acoustic research. To determine L_Aeq_ value free-field response was used. This response was chosen because this work is a part of a larger study, which is conducted in free-field conditions. Additional analysis of results including the use of diffuse-field response showed that differences in L_Aeq_ values calculated for both responses (free-field and diffuse-field) range from 0.3 to 1.3 dB and they do not significantly affect the conclusions obtained from this work.

Most of the available studies on the parameters of the sounds reproduced by headsets are based on measurements under real-life conditions. Most of these studies include the results of measurements of the L_Aeq_. Researchers less frequently include in their studies measurements of the L_Amax_ and L_Cpeak_. For example, Peretti et al. [9] conducted research on the noise exposure of telephone-central-office and call-centre staff who used headsets or headphones for their work. These measurements were carried out using a Brüel&Kjær Head and Torso Simulator Type 4128 (Hottinger Brüel&Kjær A/S, Virum, Denmark). The L_Aeq_ values obtained in these studies ranged from 50 to 87 dB, as converted for the diffuse-field frequency response (ISO 11904-2:2021 [3]). Daltrop and Bessey [10] proposed a similar solution. They measured the noise exposure of call-centre staff using a manikin. The L_Aeq_ value ranged up to 83 dB when converted according to the diffuse-field frequency response (ISO 11904-2:2021 [3]). As in the present study, Daltrop and Bessey also sought to determine L_Cpeak_ values without applying a conversion. The highest recorded L_Cpeak_ was 116 dB.

The Knowles Electronics Manikin for Acoustic Research (KEMAR) (GRAS Sound & Vibration, Holte, Denmark) was also used to assess the noise exposure of workers using headsets. This manikin model was used by Dajani et al. [11] and Patel and Groughton [12]. The L_EX,8h_ values obtained by Dajani et al. [11] were up to 80 dB for office staff and up to 95 dB for air-traffic-control staff (both of these values were converted according to the diffuse-field frequency response according to Kunov et al. [29]). By contrast, research by Patel and Groughton [12] included an analysis of the L_Aeq_ among a large group of call-centre operators. These studies showed that the L_Aeq_ values ranged from 65 to 88 dB (converted according to the free-field frequency response according to Rice et al. [30]). The results of the tests carried out in real-life conditions using different types of manikins do not differ significantly from those published in this article. The L_Aeq_ values obtained under laboratory conditions are higher than those measured under real-life conditions at call centres. This is due to the fact that the L_Aeq_, unlike the L_Cpeak_, is affected under real-life conditions by time periods in which no sound is reproduced through headsets. For measurements under laboratory conditions, there were no periods of time during which the test signal was not reproduced. However, highly consistent measurement results were obtained for the L_Cpeak_ parameter.

Apart from manikins, the available literature includes references to acoustic signal exposure tests involving headsets with miniature microphones. For instance, Chiusano et al. [13] used the Knowles Electronics BL-1785 (Knowles Electronics LLC., Itasca, IL, USA) miniature microphone to examine the noise exposure of headset users from the US Department of Defence. The authors presented the results for the L_Aeq_ and peak sound level measurements (without C-weighting). The results showed that headset users were likely to be exposed to the risk of hearing damage, because the L_Aeq_ was up to 103 dB and the peak sound level was above 140 dB. Please note that these results were not converted according to the diffuse-field frequency response.

The same type of microphone was used by Smagowska [14] in noise-exposure measurements for call-centre operators. The phone conversations conducted by the operators were recorded to measure the following noise parameters: L_Aeq_ of 68 to 91 dB, L_Amax_ of 88 to 102 dB and L_Cpeak_ of 97 to 125 dB. This is one of the few studies, in addition to this one, to examine the values of L_Aeq_, L_Amax_ and L_Cpeak._ However, no conversion was performed for the free-field or diffuse-field frequency response. Another study involving the measurement of the L_Aeq_, L_Amax_ and L_Cpeak_ for call-centre operators was conducted by Pawlaczyk-Łuszczyńska et al. [15]. In this research, a GRAS 43AG-2 (GRAS Sound & Vibration, Holte, Denmark) artificial ear was used, converting the results of the L_Aeq_ measurements with the diffuse-field frequency response in accordance with CSA Z107.56-13 [7]. The mean values of the L_Aeq_, L_Amax_ and L_Cpeak_ presented in the above study were 78, 97.2 and 115.9 dB, respectively.

Table 5 presents a summary of the highest values of the noise parameters produced by headsets or headphones determined by different measurement methods under real conditions taken from the literature and obtained in this work under laboratory conditions. It is impossible to take into account all the reported situations, but the noise parameter values obtained in this work are within the range of values encountered in real conditions. Thus, the volume control level settings adopted in this study (75%, 90% and 100%) result in SPL values produced by headsets encountered in real-world situations.

Another study that involved the use of an artificial ear in sound measurements was carried out by Williams and Presby [16]. The L_Aeq_ was measured using the method described in AS/NZS 1269.1: 1998 [31]. These measurements involved the use of a Brüel&Kjær 4152 (Hottinger Brüel&Kjær A/S, Virum, Denmark) artificial ear and a single-number conversion for the diffuse-field conditions of 8 dB. The L_Aeq_ values obtained ranged from 52 to 95 dB. Studies by Nassrallahi et al. [32] showed that the use of single-number conversion increases the uncertainty of measurements, as this conversion does not take into account the spectral nature of audio signals. The authors of these studies recommend that, for measurements taken with artificial ears, the conversion should be performed in one-third-octave frequency bands, as described in ISO 11904-2:2021 [3].

A similar conclusion can be drawn for measurements involving acoustic manikins. AS/NZS 1269.1:2005 [6] and CSA Z107.56-13 [7] specify that a single-number conversion for free-field and diffuse-field conditions should be 5 dB. The free-field related conversion values obtained in this study in accordance with ISO 11904-2:2021 [3] ranged from 2.4 to 10.8 dB around the mean value, taking into account all the measurement scenarios (different headsets, signal volume control levels, connector types, computer models and sound card types)—4.6 dB. Therefore, the mean value of the conversion was similar to that specified in AS/NZS 1269.1:2005 [6] and CSA Z107.56-13 [7]. However, the use of single-number conversion may both inflate and lower, by a few dB, the correct L_Aeq_ value of the signal reproduced by headsets.

Two different methods of measuring the parameters of the noise exposure of the workers using headsets, i.e., the artificial ear method and the miniature microphone method, were described by Pawlaczyk-Łuszczyńska et al. [17]. Noise parameter tests were carried out on a group of 74 employees, including military aviation personnel, transcribers and call-centre operators. The L_Aeq_, L_Amax_ and L_Cpeak_ values reproduced by headsets were measured using a SVANTEK SV25S (Svantek Sp. z o.o., Warsaw, Poland) miniature microphone and a GRAS 43AG-2 (GRAS Sound & Vibration, Holte, Denmark) artificial ear according to ISO 11904-1:2002 [2] and CSA Z107.56-13 [7], respectively. The measurement results were compared using both these methods and found to be highly consistent in the case of the L_Aeq_ for military aviation personnel (the maximum difference between the mean values was 0.3 dB). However, greater discrepancies were observed for transcribers and call-centre operators. The differences between the mean L_Aeq_ values for both these groups were 2.4 and 5.9 dB, respectively. Even greater discrepancies were found for the L_Amax_ and L_Cpeak_ between the results of the measurements using a miniature microphone and those with an artificial ear, i.e., up to 10 dB. Comparative studies of the standardised methods of measuring the sound generated by headsets were also carried out by Nassrallahi et al. [32]. These tests were carried out in laboratory conditions with the use of a GRAS 45BA (GRAS Sound & Vibration, Holte, Denmark) manikin, a GRAS 43AG (GRAS Sound & Vibration, Holte, Denmark) Type 3.3 artificial ear, a GRAS RA0045 (GRAS Sound & Vibration, Holte, Denmark) Type 2 artificial ear and a Brüel&Kjær 4153 (Hottinger Brüel&Kjær A/S, Virum, Denmark) Type 1 artificial ear. The researchers found that there was little consistency between the results of the measurements using a Type 1 artificial ear for audiometric calibration purposes and the results of the measurements carried out using a manikin (according to ISO 11904-2:2021 [3]). By contrast, the results of the measurements involving the Type 2 and 3.3 artificial ear and the manikin were found to be highly consistent. It can therefore be concluded that tests of the parameters of the acoustic signal reproduced by headsets can be carried out, just as in this work, using a manikin. Another option is to use an artificial ear for which highly consistent results have been achieved relative to the results of measurements obtained using a manikin. However, it is important to use the appropriate types of artificial ears for this purpose. The use of miniature microphones is also possible. Research has shown that the results of measurements taken using a miniature microphone are potentially consistent with those taken using an artificial ear. However, the use of miniature microphones is limited due to the possibility of practical problems with the positioning of microphones in the ear canal and the risk that accidental microphone movements will cause artefacts to appear during the measurements.

The analysis of the noise parameters generated by headsets included in this study confirmed that a reduction in the volume control level of the test signal results in a decrease in the L_Aeq_ values. The extent of this change depends on the model of headset used. For headsets with USB and Bluetooth connectivity, the dynamics of the L_Aeq_ changes depend on whether the model of the headset used is equipped with its own sound cards (analogue-to-digital converters). In the case of a 3.5 mm-jack connector, this dynamic depends on the type of sound card the computer uses and, to a lesser extent, on the model of the headset used. The use of a mobile audio interface led to the dynamics of the L_Aeq_ changes being greater than those in the case of the sound card being integrated into the computer motherboard.

A comparison of the L_Aeq_ values of the test signal reproduced through headsets connected to two different computers (from different manufacturers) reveals small differences between these values when a 3.5 mm-jack connector is used. When headsets were connected to a mobile audio interface, there was a significant reduction (statistically significant) in L_Aeq_ values compared to when headsets were connected directly to the integrated sound card. However, when headsets were connected to two different computers (from different manufacturers) via USB, the observed changes in L_Aeq_ values were not statistically significant. This is because the L_Aeq_ is affected by sound cards that are used in headsets rather than by sound cards integrated into the computer motherboard.

## 5. Conclusions

Analysis of the measured levels of the L_Aeq_, L_Amax_ and L_Cpeak_ showed that, for 4 out of the 12 headsets used in the tests, the values of L_Aeq_ reproduced by these headsets were higher than 85 dB. This means that there is a risk of exceeding the limit value of L_EX,8h_ [4] for headset users who are exposed to the sound for a long time and, as a consequence, there is a risk of hearing damage to the headset user. However, no evidence of exceeding the limit values [4] for the L_Amax_ and L_Cpeak_ was found.

Research has shown that the same change in the volume control level of the acoustic signal reproduction path may affect the L_Aeq_ measured with these headsets in very different ways, depending on the type of headset connector used (USB or 3.5 mm jack). The use of a 3.5 mm-jack connector rather than a USB port may cause the L_Aeq_ to remain high with these headsets, despite a reduction in the control level.

Headset users should therefore set the control level value to ensure that it is not at its maximum and avoid high levels of the acoustic signal unless necessary. Additionally, background noise such as from other equipment should be limited so that there is no need to set the acoustic signal from headsets to a high level. In addition, headsets users should have their hearing tested regularly to monitor the effects of noise. There should also be additional health and safety measures in place for employees who use headsets and work at home. This aspect of employees’ health is overlooked in common occupational safety and health measures.

## Figures and Tables

**Table 1:** Values of A-weighted equivalent sound pressure level of the test signal reproduced by various headsets that were directly connected to Laptop 1 and Laptop 2, and to Laptop 1 through a mobile audio interface.

Headset	Connector	Volume Control Level	Laptop 1	Laptop 2	Laptop 1
			Sound Card Integrated into the Motherboard	Sound Card Integrated into the Motherboard	Mobile Audio Interface
			A-Weighted Equivalent Sound Pressure Level, dB		
A	USB	100%	83.4	83.9	-
B	USB	100%	76.4	76.8	-
C	USB	100%	79.7	79.8	-
D	3.5 mm jack	100%	82.6	83.7	71.5
E	USB	100%	73.7	73.6	-
	3.5 mm jack	100%	74.9	79.3	64.2
F	USB	100%	84.3	83.7	-
	3.5 mm jack	100%	81.4	85.0	71.3
G	USB	100%	90.6	90.6	-
	3.5 mm jack	100%	86.8	92.5	77.9
		90%	85.2	91.1	-
		75%	-	88.9	-
H	USB	100%	88.5	87.7	-
	3.5 mm jack	100%	85.3	90.6	75.4
		90%	-	89.0	-
I	USB	100%	88.9	84.5	-
	3.5 mm jack	100%	87.0	89.6	75.8
		90%	85.3	88.3	-
		75%	-	86.1	-
J	Bluetooth	100%	88.3	87.7	-
		90%	85.9	85.1	-
K	USB	100%	80.4	80.0	-
L	USB	100%	81.3	81.1	-

**Table 2:** Values of A-weighted maximum sound pressure level of the test signal reproduced by various headsets that were directly connected to Laptop 1 and Laptop 2, and to Laptop 1 through a mobile audio interface.

Headset	Connector	Laptop 1	Laptop 2	Laptop 1
		Sound Card Integrated into the Motherboard	Sound Card Integrated into the Motherboard	Mobile Audio Interface
		A-Weighted Maximum Sound Pressure Level, dB		
A	USB	93.4	93.8	-
B	USB	87.8	87.8	-
C	USB	89.4	89.2	-
D	3.5 mm jack	94.8	95.1	84.4
E	USB	84.9	84.8	-
	3.5 mm jack	87.3	90.2	76.2
F	USB	93.3	93.1	-
	3.5 mm jack	91.5	94.8	83.0
G	USB	97.5	97.0	-
	3.5 mm jack	93.1	99.8	86.3
H	USB	95.3	94.5	-
	3.5 mm jack	91.9	97.9	84.0
I	USB	96.2	92.3	-
	3.5 mm jack	93.2	97.4	84.5
J	Bluetooth	95.1	94.4	-
K	USB	91.2	91.0	-
L	USB	94.0	93.7	-

**Table 3:** Values of C-weighted peak sound pressure level of the test signal reproduced by various headsets that are directly connected to a Laptop 1 and a Laptop 2, and to a Laptop 1 through a mobile audio interface.

Headset	Connector	Laptop 1	Laptop 2	Laptop 1
		Sound Card Integrated into the Motherboard	Sound Card Integrated into the Motherboard	Mobile Audio Interface
		C-Weighted Peak Sound Pressure Level, dB		
A	USB	113.6	114.1	-
B	USB	109.0	109.2	-
C	USB	110.3	110.4	-
D	3.5 mm jack	114.2	115.1	104.7
E	USB	106.5	106.4	-
	3.5 mm jack	107.5	110.5	96.1
F	USB	114.1	114.4	-
	3.5 mm jack	111.0	115.2	103.6
G	USB	114.4	113.8	-
	3.5 mm jack	110.9	116.6	105.6
H	USB	112.3	111.1	-
	3.5 mm jack	110.1	114.8	104.0
I	USB	113.8	111.1	-
	3.5 mm jack	111.0	114.8	104.2
J	Bluetooth	113.2	113.3	-
K	USB	112.9	112.4	-
L	USB	114.8	114.5	-

**Table 4:** Changes in the test signal value due to changes in volume control level.

Type of Measurement	Source of Signal	Change in Volume Control Level of Test Signal				
		100–90%	90–75%	100–75%	75–50%	50–25%
		Change in the Test Signal Value, dB				
Acoustic	Laptop 1	1.6	2.3	-	-	-
	Laptop 2	1.4	2.3	-	-	-
	Mobile audio interface	4.5	7.5	-	-	-
Electric	Laptop 1	1.4	2.1	3.5	6.0	10.5
	Laptop 2	1.5	2.3	3.8	6.0	10.5
	Mobile audio interface	4.5	7.5	12.0	12.0	11.7

**Table 5:** A summary of the highest values of L_Aeq_, L_Amax_ and L_Cpeak_ determined by different measurement methods under real conditions taken from the literature and obtained in this work under laboratory conditions.

Reference	Measurement Conditions	Highest Values of Measured Noise Parameters		
		A-Weighted Equivalent Sound Pressure Level, dB	A-Weighted Maximum Sound Pressure Level, dB	C-Weighted Peak Sound Pressure Level, DB
Peretti et al. [9]	Telephone central office and call centre	87		
Daltrop and Bessey	Call centre	83		116
Dajani et al. [11]	Office and air traffic control	80 and 95 *		
Patel and Groughton [12]	Call centre	88		
Chiusano et al. [13]	US Department of Defense	103		140 **
Smagowska [14]	Call centre	91	102	125
Pawlaczyk-Łuszczyńska at al. [15]	Call centre	78 ***	97.2 ***	115.9 ***
Williamsa and Presburego [16]	Radio announcers	95		
This paper	Laboratory	92.5	97.5	116.6

* This work did not strictly determine the value of L_Aeq_. Instead, the L_EX,8h_ values are given, which were determined from the L_Aeq_ values and exposure duration. ** This work did not determine the value of L_Cpeak_. Instead, the L_peak_ values are given, which are slightly higher than the values obtained using the C-weighting. *** This work did not determine the highest value of L_Aeq_, L_Amax_ and L_Cpeak_. Instead, the total mean values of these parameters are given.

## Data Availability

All the data are stored digitally by the researcher. The 1/3-octave band data are available on request from the corresponding author.
